# Effect of blood pressure threshold on adverse outcomes in patients with acute spinal cord injury: a systematic review and meta-analysis

**DOI:** 10.1186/s13054-025-05531-3

**Published:** 2025-07-16

**Authors:** Azasma Tanvir, Abramo Aziz Rizk, Wendy Wang, Hamed Alsagga, Husain Shakil, Ha Le, Prosanta Mondal, Frederick A. Zeiler, Tumul Chowdhury

**Affiliations:** 1 Department of Anesthesiology, University of Saskatchewan, Saskatoon, Canada; 2 Faculty of Health Sciences, McMaster University, Hamilton, Canada; 3 Temerty Faculty of Medicine, University of Toronto, Toronto, Canada; 4 Department of Anesthesia and Pain Medicine Toronto Western Hospital, Clinician Investigator, UHN, University of Toronto, Toronto, Canada; 5 Division of Neurosurgery, Department of Surgery, University of Toronto, Toronto, Canada; 6 Clinical Research Support Unit, College of Medicine, University of Saskatchewan, Saskatoon, Canada; 7 Section of Neurosurgery, Department of Surgery, Rady Faculty of Health Sciences, University of Manitoba, Winnipeg, Canada; 8 Biomedical Engineering, Price Faculty of Engineering, University of Manitoba, Winnipeg, Canada; 9 Pan Am Clinic Foundation, Winnipeg, Canada

**Keywords:** Spinal cord injury, Functional recovery, Blood pressure, Mean arterial pressure, Vasopressors, Meta-analysis

## Abstract

**Background:**

Inadequate spinal perfusion in acute spinal cord injury (SCI) can exacerbate secondary injury. While current guidelines recommend maintaining mean arterial pressure (MAP) ≥ 75-80mmHg post-injury, no quantitative analysis on effects of blood pressure on neurological outcomes exists. We aim to address this gap and evaluate the impact of blood pressure thresholds on adverse outcomes in acute traumatic and non-traumatic SCI to inform current guidelines.

**Methods:**

The project adhered to PRISMA and MOOSE guidelines and was registered in PROSPERO (CRD42024550044). We searched seven databases: MEDLINE, Embase, Cochrane Central, Cochrane Reviews, CINAHL, Scopus, and Web of Science. We included studies involving patients ≥ 16yrs with acute SCI, randomized control trials, prospective cohorts, and retrospective (case-control, cohort) studies. Excluded were chronic SCI and studies mentioning induced hypotension. The main outcome was the relationship between blood pressure thresholds and adverse functional outcome at up to one-year post-injury. Outcomes (unadjusted odds ratios (uOR) and adjusted odds ratios (aOR)) were calculated using a random-effect model with 95% confidence intervals (CI). Quality was assessed using the Newcastle-Ottawa Scale and Cochrane Risk of Bias Tool.

**Results:**

Of 16,366 identified articles, 38 (*n* = 7,167, 73% male) were included in the qualitative and 14 (*n* = 2,553, 76% male) in the quantitative analysis. Pooled analysis found an increase in adverse functional outcomes in patients with below threshold blood pressures (uOR, 3.28; 95% CI, 2.39–4.50; aOR, 1.04; 95% CI, 1.03–1.05). Subgroup analyses consistently showed that lower blood pressure thresholds were associated with worse outcomes across all subgroups. Risk of bias was low to moderate in most studies. Heterogeneity was moderate to high (I^2^: 69.88%).

**Conclusion:**

Lower blood pressure thresholds were consistently associated with worse functional outcomes in patients with acute SCI. While these findings support the rationale for MAP augmentation, they should be interpreted cautiously due to the observational nature of the data and high heterogeneity. High-quality prospective studies are needed to determine optimal blood pressure targets.

**Supplementary Information:**

The online version contains supplementary material available at 10.1186/s13054-025-05531-3.

## Background

Spinal cord injury (SCI) is a prevalent issue worldwide, with the World Health Organization (WHO) estimating around 15.4 million people living with SCI in 2021 [1]. Hypotension, a common complication in patients with SCI, has been found to precipitate secondary injury, due to ischemia and hypoxia, leading to worse clinical outcomes [2]. The 2024 AO Spine & Praxis guidelines recommend maintaining mean arterial pressure (MAP) of at least 75–80 mmHg for the first 3–7 days to optimize spinal cord perfusion in acute traumatic SCI [3]. However, the quality of evidence supporting this recommendation is very low, and the strength of the recommendation is weak. Moreover, there is an unmet need for quantitative analyses examining the effect of blood pressure (BP) thresholds on outcomes for patients sustaining SCI. Our study aimed to address this gap by reviewing relevant literature and presenting the first meta-analysis pertaining to the impact of low BP thresholds on adverse functional outcomes in patients with SCI.

## Methods

This review is registered in the International Prospective Register of Systematic Reviews (PROSPERO) (CRD42024550044) and follows the Preferred Reporting Items for Systematic Review and Meta-Analyses (PRISMA) [4] and Meta-analysis of Observational Studies in Epidemiology (MOOSE) [5] (Supplement eTables 11–12) reporting guidelines.

### Data sources

The search strategy was developed in collaboration with an institutional librarian. Literature searches were conducted from inception until June 10, 2024, across the following databases: (1) MEDLINE, (2) Embase, (3) Cochrane Central Register of Controlled Trials, (4) Cochrane Database of Systematic Reviews, (5) Cumulative Index to Nursing & Allied Health Literature (CINAHL), (6) Web of Science, and (7) Scopus. The MEDLINE search strategy is provided in Supplement eTable 1.

### Study selection

Initial title and abstract screening was performed by multiple independent reviewers (AT, AAR, WW, HA). Full text for titles that passed initial screening were retrieved and screened for eligibility. Disagreements were resolved by consensus or by consulting a senior author (TC). Inter-reviewer reliability was assessed using Cohen’s Kappa, which measures the degree of agreement between reviewers. The selection process was conducted using the Covidence software [6]. 

The inclusion criteria were: (1) original research articles investigating BP thresholds in patients with SCI; (2) population: patients ≥ 16 years presenting with acute traumatic or non-traumatic SCI; (3) intervention: any type of BP threshold; (4) study design: primary research articles including randomized control trials (RCTs), prospective cohorts, and retrospective (case-control, cohort) studies. Exclusion criteria were: (1) abstracts, case series, case reports, reviews, letters, post hoc analyses, editorials; (2) articles discussing controlled or induced hypotension. No language restriction was applied. Only studies reporting odds ratios (ORs) or allowing calculation of ORs from study data were included in the meta-analysis.

### Data extraction and synthesis

Summary data was extracted by four authors (AT, AAR, WW, HA) using a standardized data collection spreadsheet, including study characteristics, patient characteristics (age, sex, injury severity and mechanism), sample size, spinal level, BP threshold, functional outcomes, and study quality. All data was extracted from published reports, and study authors were contacted if additional information was required.

The primary outcome of interest was the functional outcome at up to one year post injury, defined as either neuromotor recovery (measured by the American Spinal Injury Association Scale (ASIA) grade recovery) or visceral organ function (defined as internal organ function, such as bladder, bowel, and respiratory function). We focused on functional outcomes as these were most often reported across studies and therefore most suitable for a meta-analysis. Secondary outcomes included vasopressor usage. We extracted ORs reported for the relationship between BP thresholds and functional outcomes. If an OR was not provided, we calculated it using the study’s exposure (patients above or below the BP threshold) and adverse outcome (binary) if possible. Among studies reporting only correlation coefficients, we estimated the corresponding OR as described previously in the literature [7, 8]. 

Quality assessments of all included studies were performed by two reviewers (AAR, HA) using the Newcastle-Ottawa Scale (NOS) for cohort studies. The quality of studies was based on total points, with 7–9 points for good quality studies, 4–6 points for moderate quality, and 1–3 points for poor quality [9]. The Cochrane Risk of Bias Assessment was applied for RCTs [10]. We evaluated the certainty of evidence using the Grading of Recommendations Assessment, Development, and Evaluation (GRADE) [11] framework approach, which considers several factors: risk of bias, indirectness, imprecision, inconsistency, and potential publication bias.

### Statistical analysis

Meta-analysis was performed using Stata software version 18.0 (Stata Corporation, College Station, Texas). The measures of effect of interest were unadjusted OR (uOR) and adjusted OR (aOR) with 95% confidence intervals (CI). Adjusted effect estimates, when available, were extracted as reported by the individual studies, which includes adjustment for covariates including age, injury severity, sex, and comorbidities (Supplement eTable 3). Subgroup analyses were conducted to explore potential sources of heterogeneity. The statistical heterogeneity between and within groups was assessed using Cochran’s Q statistic. A p-value < 0.05 was considered statistically significant. We calculated pooled estimates and CIs assuming a random-effects model. Sensitivity analysis was performed using a leave-one-out analysis. Publication bias was investigated both visually by using a funnel plot and statistically with Egger’s bias test, which measures the degree of funnel plot asymmetry. *P* < 0.1 was considered to be representative of statistically significant publication bias.

## Results

### Search results and study characteristics

**Fig. 1:**
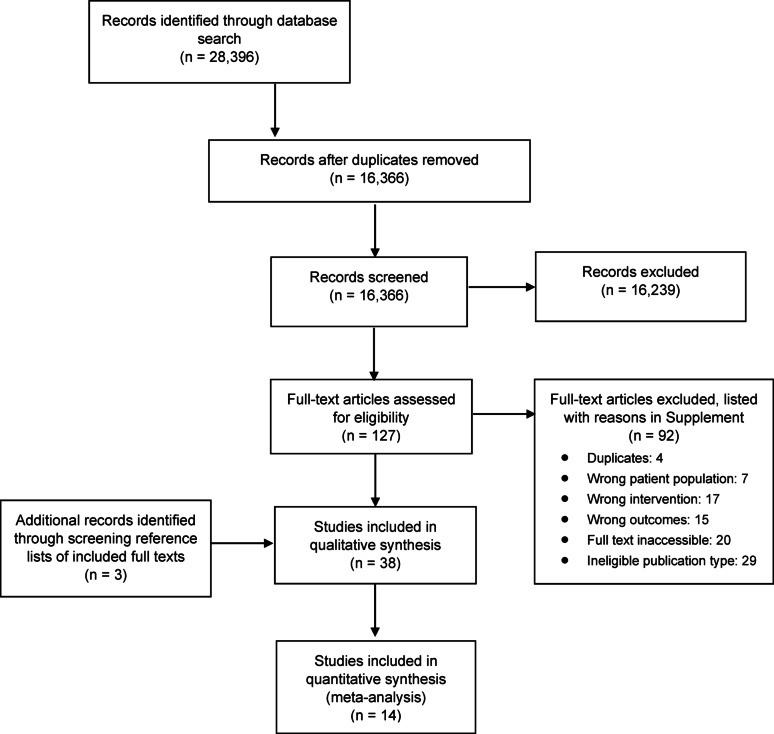
PRISMA flow diagram of study selection

The search identified 28,396 studies. After removing duplicates, 16,366 were screened for title and abstracts, of which 127 were included for full text screening. Of these, 92 were excluded, with reasons listed in Fig. 1 and Supplement eTable 9. The Cohen’s Kappa value for full text screening was 0.97, indicating excellent agreement between reviewers. Three additional articles were identified from citation searching, resulting in a total of 38 articles included for qualitative analysis [12–49]. Of these, 14 were eligible for quantitative meta-analysis [13, 20, 21, 25, 26, 29–32, 41, 43, 44, 46, 47] as they either reported ORs directly or provided sufficient data to allow OR calculation as outlined in the methods section.

The demographics and study characteristics of the 38 included studies are summarized in Table 1. A total number of 7,167 participants [mean age (std) 48.17 (14), 73% male] from 38 studies were included. Twelve studies (31%) were prospective cohort [18, 23, 27–29, 33, 35, 42, 43, 45, 46, 48], 22 (58%) were retrospective cohort [12, 13, 15–17, 19–22, 24–26, 31, 32, 34, 36–40, 44, 47], 3 (8%) were retrospective case control [14, 30, 49], and one (3%) was an RCT [41]. 

**Table 1:** Study characteristics of all included studies

Study	Country	Study design		Sample size		Mean age		Male (%)		SCI severity		SCI scale		Type of blood pressure		BP threshold (mmHg)		BP measurement setting		Days followed		Type of SCI	
Agarwal [12]	USA	RC		74		51		73		Mod, Sev		AISA		MAP		76		Intraoperative		Discharge		Blunt/ Penetrating tSCI	
Alfin [13]	Nigeria	RC		296		35.96		83.3		Mod, Sev		ASIA		MAP		80		Neurosurgical unit		42		Blunt tSCI	
Balasuberamaniam [14]	Canada	RCC		82		50.6		89		Sev		AISA		MAP		80		Trauma unit		Discharge		tSCI	
Blue [15]	USA	RC		910		61.6		48.9		NA		NA		MAP		65		Intraoperative		NA		Intraoperative trauma	
Catapano [16]	USA	RC		62		41.6		NA		Sev		ASIA		MAP		85		ICU		40		Blunt/ Penetrating tSCI	
Chen [17]	China	RC		105		48.78		68.6		Mod, Sev		ASIA		SBP		NA		On admission, ICU		180		Cervical tSCI	
Chen [18]	UK	PC		45		NA		77.8		Sev		ASIA		SCPP		NA		ICU		270–365		Blunt tSCI	
Chiang [19]	China	RC		1833		NA		57.4		NA		NA		SBP		80		Intraoperative		NA		Intraoperative trauma	
Cohn [20]	USA	RC		17		32.8		82.4		Sev		ASIA		MAP		Variable		In hospital		7		Blunt tSCI	
Dakson [21]	Canada	RC		94		47.6		79		Mod, Sev		ASIA		MAP		85		ICU		26.7		Blunt tSCI	
Ehsanian [22]	USA	RC		25		42.44		68		Mod, Sev		ASIA		MAP		50		Intraoperative		41.8		Blunt tSCI	
Gallagher [23]	UK	PC		12		40		66.7		Sev		ASIA		SCPP		NA		Neurosurgical unit		240		Blunt tSCI	
Glassman [24]	USA	RC		539		59.8		42		NA		NA		MAP		65		Intraoperative		30		Intraoperative trauma	
Haldrup [25]	Denmark	RC		129		50		81.4		Mod, Sev		ASIA		MAP		80		Pre-Hospital, Intraoperative, ICU		365		Blunt tSCI	
Hawryluk [26]	USA	RC		100		NA		75.3		Mod, Sev		ASIA		MAP		85		ICU		30		Blunt/Penetrating tSCI	
Hogg [27]	UK	PC		14		47.4		92.9		Sev		ASIA		SCPP		60		ICU		243		Blunt tSCI	
Hogg [28]	UK	PC		13		47.1		92.3		Sev		ASIA		SCPP		60		ICU		365		Blunt tSCI	
Jiang [29]	Canada	PC		798		44.6		78		Mod, Sev		AISA		MAP, SBP		85, 90		On admission		365		Blunt/Penetrating tSCI	
Kamel [30]	USA	RCC		152		53		53		Mod, Sev		NA		MAP		55		Intraoperative		NA		Intraoperative trauma	
Långsjö [31]	Finland	RC		51		54.9		84		Mod, Sev		ASIA		MAP		85		ICU		110, 69		Cervical tSCI	
LaRiccia [32]	USA	RC		96		52.74		77		Mod, Sev		ASIA		MAP		85		Trauma unit		NA		Blunt tSCI	
Li [33]	China	PC		102		63		42.2		NA		ASIA		MAP		Variable		Intraoperative		NA		Intraoperative trauma	
Martin [34]	USA	RC		105		49.53		70.5		Mod, Sev		AMS		MAP		85		ICU		Discharge		Blunt/Penetrating tSCI	
Müller [35]	Germany	PC		99		72.2		50.5		NA		NA		SBP		NA		Intraoperative		365		Intraoperative trauma	
Mushlin [36]	USA	RC		193		43		82		Sev		ASIA		MAP		85		Neurosurgical unit		180		Cervical tSCI	
Rask [37]	Sweden	RC		52		53		73		Mod, Sev		ASIA		MAP		85		ICU		90		Blunt tSCI	
Readdy [38]	USA	RC		36		39.39		77.8		Sev		ASIA		MAP		85		ICU		Discharge		Penetrating tSCI	
Rerikh [39]	Russia	RC		104		34.3		82.7		Mod, Sev		ASIA		MAP		85		ICU		120–180		Blunt tSCI	
Santos [40]	Brazil	RC		49		34.7		89.8		Mod, Sev		ASIA		SBP		90		Trauma unit		Discharge		Blunt/Penetrating tSCI	
Sharma [41]	India	RCT		60		55.45		NA		NA		NA		MAP		NA		Intraoperative		365		Intraoperative trauma	
Squair [42]	Canada	PC		92		43		78.3		Sev		ASIA		MAP, SCPP		80, 60		ICU		180		NA	
Squair [43]	Canada	PC		92		43		78.3		Sev		ASIA		MAP, SCPP		80, 50		ICU		180		NA	
Tee [44]	Australia	RC		570		49.2		71.9		Mod, Sev		ISS		SBP		100		Trauma unit		365		Blunt tSCI	
Visagan [45]	UK	PC		13		44.17		76.9		Sev		ASIA		MAP		85		ICU		237		Cervical tSCI	
Vale [46]	USA	PC		64		NA		84		Mod, Sev		ASIA		MAP		85		ICU		365		Blunt tSCI	
Weinberg [47]	USA	RC		136		52		75		Mod, Sev		ASIA		MAP		85		Trauma unit		NA		Blunt tSCI	
Werndle [48]	UK	PC		18		45.16		61.2		Sev		ASIA		SCPP		60		ICU		7		Blunt tSCI	
Zhang [49]	China	RCC		40		54.67		67.5		Mod, Sev		ASIA		MAP		70		On admission		365		Cervical tSCI	

ASIA = American Spinal Injury Association Scale; BP = Blood Pressure; ICU = Intensive Care Unit; MAP = Mean Arterial Pressure; Mod = Moderate; NA = Not Available; PC = Prospective Cohort; RC = Retrospective Cohort; RCC = Retrospective Case Control; RCT = Randomized Control Trial; SBP = Systolic Blood Pressure; SCI = Spinal Cord Injury; SCPP = Spinal Cord Perfusion Pressure; Sev = Severe; tSCI = Traumatic Spinal Cord Injury

Meta-analysis of 14 studies (2,553 patients) revealed that lower BP thresholds (as defined in the individual study, based on the 2013 American Association of Neurological Surgeons (AANS) guidelines) was associated with increased adverse functional outcomes (uOR: 3.28, 95% CI: 2.39–4.50, *p* < 0.0001; aOR:1.04, 95% CI: 1.03–1.05, *p* < 0.0001) (Supplement eFigure 1).

**Fig. 2:**
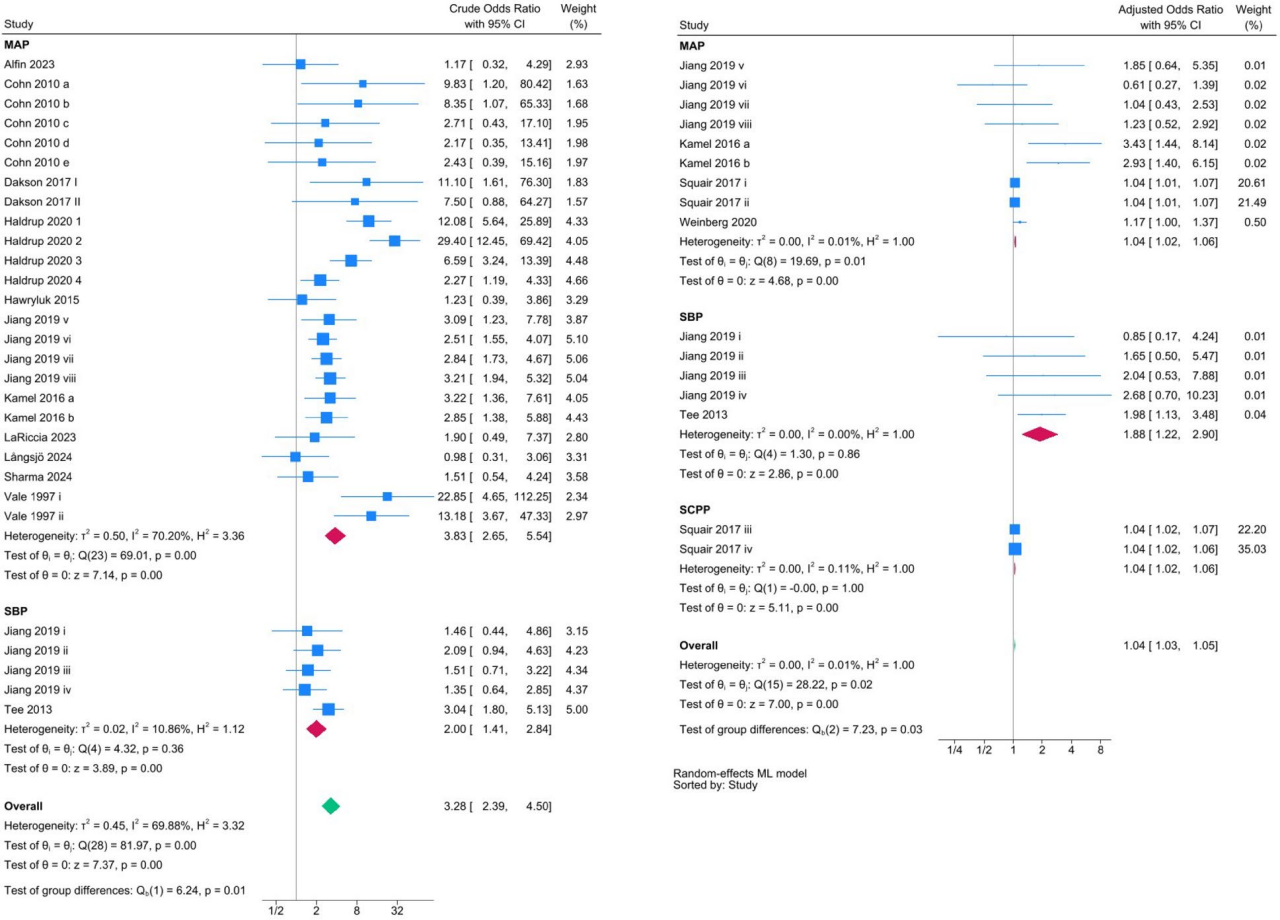
Forest plot summarizing the unadjusted (left) and adjusted (right) odds ratios for adverse outcomes associated with blood pressure thresholds in acute SCI

Subgroup analyses assessing MAP and SBP thresholds found that studies reporting both MAP and SBP thresholds showed an association between lower BP and adverse functional outcomes. The uORs for MAP and SBP thresholds were 3.83 (95% CI: 2.65–5.54, *p* < 0.0001) and 2.00 (95% CI: 1.41–2.84, *p* < 0.0001), respectively. The aORs were 1.88 (95% CI: 1.22–2.90, *p* < 0.0001) and 1.04 (95% CI: 1.02–1.06, *p* < 0.0001), respectively (Fig. 2).

In a subgroup analysis comparing MAP thresholds, patients whose MAP fell below 85 mmHg had higher odds of adverse functional outcome as compared to those whose MAP was maintained at or above 85 mmHg. This directionality was noted for both uOR [4.86 (95% CI: 2.73–8.64, *p* < 0.0001) vs. 2.85 (95% CI: 2.26–3.60, *p* < 0.0001)] and aOR [1.54 (95% CI: 0.94–2.52, *p* = 0.09) vs. 1.15 (95% CI: 0.99–1.34, *p* = 0.06)] (Supplement eFigure 3).

To assess the association between BP thresholds and adverse functional outcomes based on severity of SCI, as measured by the ASIA Impairment Scale, studies were grouped into two categories: complete motor complete injury (ASIA A and B) in 1) ≥ 50% of patients and 2) < 50% of patients. Pooled uOR for the two groups was 7.27 (95% CI: 2.35–22.48, *p* < 0.0001) and 2.01 (95% CI: 1.18–3.43, *p* = 0.01), respectively (Supplement eFigure 4).

The effects of lower BP thresholds were further analyzed based on the level of SCI. For both cervical (uOR: 2.71, 95% CI: 2.13–3.47, *p* < 0.0001) and thoracolumbar SCI (uOR: 3.00, 95% CI: 1.72–5.22, *p* < 0.0001), lower BP thresholds had higher odds of adverse functional outcome (Supplement eFigure 5).

Subgroup analysis for studies reporting only isolated SCI and studies reporting both isolated and polytraumatic SCI showed an uOR of 3.15 (95% CI: 2.23–4.46, *p* < 0.0001) and aOR of 1.04 (95% CI: 1.03–1.05, *p* < 0.0001) for isolated only injuries and an uOR of 3.89 (95% CI: 1.83–8.26, *p* < 0.0001) and aOR of 1.22 (95% CI: 1.04–1.42, *p* = 0.01) for both isolated and polytraumatic injuries (Supplement eFigure 6).

Subgroup analysis based on SCI etiology was also conducted, looking at traumatic (blunt or penetrating injury) (uOR 3.43, 95% CI: 2.40–4.89, *p* < 0.0001; aOR: 1.04, 95% CI: 1.03–1.05, *p* < 0.0001) and non-traumatic SCI (i.e. intraoperative ‘trauma’ or myelopathy) (uOR 2.57, 95% CI: 1.58–4.19, *p* < 0.0001; aOR 3.13, 95% CI: 1.78–5.50, *p* < 0.0001) (Supplement eFigure 7).

Subgroup analyses are presented for mean arterial pressure (MAP), systolic blood pressure (SBP), and spinal cord perfusion pressure (SCPP). Overall pooled estimates and heterogeneity (I²) are shown for each group.

We also separated the functional outcomes into neuromotor recovery (uOR: 3.61, 95% CI: 2.43–5.38, *p* < 0.0001; aOR: 1.04, 95% CI: 1.03–1.05, *p* < 0.0001) and visceral function (uOR: 2.54, 95% CI: 1.81–3.56, *p* < 0.0001; aOR 1.42, 95% CI: 0.91–2.23, *p* = 0.12) (Supplement eFigure 8).

Across the 38 studies, different vasopressors were used to maintain hemodynamic stability, including norepinephrine (21 studies) [14, 19, 21, 22, 24–28, 31, 32, 35, 37, 39, 40, 42, 43, 45–48], dopamine (13 studies) [12, 14, 16, 20–22, 26, 38–40, 42, 43, 46], phenylephrine (12 studies) [12, 14, 16, 20, 22, 25, 26, 33, 38, 42, 43, 47], ephedrine (2 studies) [19, 22], vasopressin (2 studies) [14, 22], dobutamine (2 studies) [37, 39], and epinephrine (1 study) [14] (Supplement eTable 2).

Dopamine was associated with an increased risk of cardiac complications (e.g., arrhythmias), whereas the risk was lower for norepinephrine and phenylephrine [12, 16, 38]. Two studies noted that vasopressor use in general was linked to cardiac dysrhythmias [18, 26]. One paper found that patients requiring vasopressors experienced a significantly increased risk of acute kidney injury (AKI) [19]. Another reported that vasopressor use was associated with an increased risk of in-hospital complications, such as pneumonia, deep vein thrombosis, unplanned return to the operating room, and unplanned intubation [47]. They also noted that higher cumulative doses of vasopressors did not improve outcomes, and the benefits of MAP augmentation diminished at excessive doses. Few studies reported outcome differences between the various vasopressors. Twelve studies maintained or monitored the BP for seven or more days [13, 18, 20, 27, 28, 36, 39, 42, 43, 45, 46, 48], three studies for five days [14, 21, 26], and five studies for three days or less [16, 17, 34, 44, 47]. 

The Galbraith plot (Supplement eFigure 10) was examined to assess potential outliers. A leave-one-out analysis was conducted (Supplement eTable 7–8) and did not reveal any studies that disproportionately influenced the overall results, supporting the robustness of the findings. There was very weak evidence of publication bias as assessed using both visual inspection via funnel plot (Supplement eFigure 9) and quantitatively via Egger’s test (z-test statistic of 0.99, *p* = 0.32). There was substantial heterogeneity across the included studies, as reflected by an I^2^ value of 69.88% (Supplement eFigure 1).

Quality assessment by the NOS (Supplement eTable 4) showed 35 studies were of ‘good’ quality, while three were ‘fair’. Cochrane Risk of Bias Assessment (Supplement eTable 5) for the RCT was found to have a low risk of bias. GRADE classification (Supplement eTable 10) identified the evidence of nine results as ‘low’ and four as ‘moderate’.

## Discussion

This meta-analysis encompassing 2,553 patients across 14 studies provides the first quantitative synthesis of the association between BP thresholds and functional outcomes following acute SCI. Our findings show a consistent association between lower BP thresholds (e.g., MAP < 85 mmHg) and worse functional recovery. This directionality was consistent across all subgroups. However, it is important to emphasize that these findings are based on observational data and do not establish causality. Whether actively targeting high MAP improves outcomes remains uncertain.

The discrepancy between the adjusted and unadjusted effect estimates in our meta-analysis is notable and likely reflects the influence of confounding variables in observational studies. Interpretation of this result is challenging due to the substantial heterogeneity across studies and relatively small number of studies reporting adjusted effect estimates. However, the consistent directionality of effect across both measures supports the underlying association between lower BP thresholds and adverse functional outcomes. Future studies with rigorous adjustments for confounders will shed light on the clinical significance of this relationship.

Our findings support the idea that a universal MAP target may offer neurological benefit but also raise questions about whether a single MAP target is appropriate for all patients. For instance, studies with a greater proportion of motor complete injuries (ASIA A-B) showed a stronger association between lower MAP and adverse outcomes. It is possible that patients with more severe injuries are more vulnerable to suboptimal perfusion, potentially due to impaired autoregulatory mechanisms [50]. A systematic review involving animal models found that the rate of axonal degeneration was higher in severe injuries [51], which may increase susceptibility to secondary ischemic damage especially in the setting of suboptimal perfusion. However, this observation needs to be validated in controlled prospective studies.

Moreover, the widespread reliance on vasopressors to augment MAP introduces clinical issues. Cumulative vasopressor exposure was reported to increase adverse complications, such as AKI and respiratory complications [18, 47]. Notably, some studies in our review observed diminishing returns or even harm with aggressive augmentation strategies. These findings pose an important question as to when vasopressor therapy becomes detrimental rather than beneficial. Determining this inflection point is essential to ensuring that interventions intended to preserve spinal cord perfusion do not inadvertently contribute to secondary morbidity. Additionally, the overall impact of vasopressors on long-term neurological recovery remains unclear. Future studies should investigate whether specific vasopressor agents contribute to improved functional outcomes or introduce additional risks. Future studies should also investigate optimal agent, dosing, and upper hemodynamic target limits for vasopressor use.

These results are consistent with results of prior reviews exploring hemodynamic management in SCI. Qualitative analyses done by Evaniew et al. [52] identified a positive association between SCPP and neurological outcomes, and Lee et al. [53] found that MAP > 85 mmHg improved outcomes. Our study builds upon prior reviews by incorporating a larger sample size and conducting extensive meta-analysis to produce an estimate of effect size. Across all reviews, limitations such as low-quality evidence, absence of meta-analyses, and study heterogeneity highlight the need for robust, prospective research to establish evidence-based protocols. These prior reviews are summarized in Table 2.

Our findings are also consistent with broader neurocritical care evidence, such as in traumatic brain injury, where hypotension has also been linked to worsened outcomes [54]. These parallels highlight the susceptibility of both the brain and spinal cord to ischemic insults and support the principle that maintaining perfusion may mitigate secondary injury and improve outcomes.

Our study also aligns with evidence that early physiologic optimization in the acute phase of SCI is associated with improved neurological outcomes. A recent meta-analysis looking at surgical decompression to restore spinal cord blood flow reported that early decompression significantly improved recovery compared to delayed surgery [55]. The report reinforces the critical nature of the early post-injury window and highlights the importance of restoring and maintaining spinal cord perfusion in neurological recovery.

**Table 2:** Comparison of existing reviews on blood pressure management and outcomes in spinal cord injury

	No. of Studies	Sample Size	Main Findings	Strengths	Limitations
Evaniew et al. [52]	• 28 studies (1 RCT, 11 cohort, 15 case series, 1 case report)	• 1433 patients	• Increased SCPP may be associated with improved neurological outcomes	• Systematic approach (PRISMA guidelines) • Recent studies included	• Heterogenous studies • Low quality evidence • No meta-analysis*
Lee et al. [53]	• 8 studies (5 prospective, 3 retrospective)	• 431 patients	• MAP > 85 mmHg benefits outcomes, but requires vasopressors, which have complications • SCPP also critical to consider	• Comprehensive review of MAP and SCPP • Vasopressors analyzed	• Single database search • No systematic review/meta-analysis*
Bertram-Ralph & Horner [56]	• 12 studies	• Unknown	• High MAP helps early SCI stages, but induced HTN is not best practice due to risks	• Discussed 2 systematic reviews with high-quality evidence	• No systematic approach (shortcut review) • No qualitative synthesis of included articles • No meta-analysis*
Sabit et al. [57]	• 9 cohort studies (2 prospective, 7 retrospective)	• 632 patients	• Objective outcomes related to MAP documented, but no clear conclusions	• Systematic approach (PRISMA guidelines) • Comprehensive database search & manual reference checks	• Small, mostly retrospective studies • Heterogenous studies • No meta-analysis*
Saadeh et al. [58]	• 11 cohort studies (2 prospective, 9 retrospective)	• 788 patients	• MAP 85–90 mmHg for 5–7 days suggested for acute SCI	• Systematic approach (PRISMA guidelines) • Vasopressors analyzed	• Lack of high-quality data • Reliance on retrospective studies • No meta-analysis*
Ploumis et al. [59]	• 7 human studies	• 906 patients	• Complete cervical SCI requires vasopressors more than other SCI • No single vasopressor proven superior	• Systematic approach (PRISMA guidelines) • Animal & human studies • Vasopressors analyzed	• Outdated (2010) • Limited database search • Low quality evidence • No meta-analysis*

*Prior reviews did not include a meta-analysis, highlighting a key distinction between these reviews and the present study, which is the first to perform a meta-analysis on this topic

A key strength of our review is its robust methodology, including a comprehensive search strategy across multiple major databases without any language restrictions. Furthermore, our study is the first to include a quantitative analysis on the relationship between BP thresholds and functional outcomes, providing a unique insight into managing SCI. We also report extensive subgroup analyses, stratifying by BP type and magnitude, spinal level, polytrauma injuries, and mechanism of injury.

Despite the strengths, there are several limitations in our study that must be acknowledged. First, the substantial heterogeneity of the included studies poses a challenge in interpreting the results. Differences in study design, patient populations, and BP management protocols may have contributed to variations in reporting outcomes, limiting the generalizability of our findings. However, the consistency in the direction of effect across all subgroup analyses lends support to the overall findings.

Second, we did not have access to individual patient data, and pooling and adjusting was done at the study level. This may increase uncertainty around effect estimates. Individual level data would allow for more accurate adjustment of confounders and stratified analyses across a broader range of variables.

Third, although we conducted subgroup analyses based on ASIA grade groupings (i.e. proportion of patients with motor complete ASIA A and B injuries), we were unable to analyze the impact of BP thresholds on each individual ASIA grade (A through E) due to limited available data. Ideally, such stratification would provide more precise insights into how injury severity changes the effect of hemodynamic management.

Finally, reliance on observational studies means that causal relationships between BP thresholds and functional outcomes cannot be definitively established. The majority of the studies in our review were retrospective in nature, which introduces the potential for confounding, selection and reporting biases, although most included studies were rated as ‘good’ based on the NOS scale. Only one included study was an RCT [41], however, its findings were broadly consistent with the overall direction observed in the observational studies, suggesting an association between higher MAP targets and improved functional outcomes.

While our study reports the first meta-analysis of the effect of BP thresholds in SCI, several research gaps remain. Future research should aim to address the limitations identified in our review by conducting well designed prospective studies to more definitively establish optimal BP targets for patients with SCI.

## Conclusion

Taken together, the evidence presented in this meta-analysis suggests a consistent association between lower BP thresholds and worse functional outcomes in patients with acute SCI. While these findings reinforce the rationale behind current guidelines recommending MAP augmentation, they should be interpreted with caution due to the observational nature of the data. Causality cannot be inferred, and it remains unclear whether targeting higher MAP values through interventions such as vasopressors would definitively improve outcomes. Given the potential risks associated with vasopressor use, future RCTs are essential to evaluate the safety and efficacy of specific hemodynamic targets in this population. Ultimately, these findings can be used to shape future investigations aimed at optimizing hemodynamic management in SCI.

## Electronic supplementary material

Additional file 1. Supplementary Appendix: Contains all supplementary information and data as referenced in manuscript.

## Data Availability

The datasets used and/or analysed during the current study are available from the corresponding author on reasonable request.

## Abbreviations

AKI: Acute kidney injury
aOR: adjusted Odds ratio
ASIA: American Spinal Injury Association Scale
BP: Blood pressure
CI: Confidence interval
GRADE: Grading of Recommendations Assessment, Development, and Evaluation
MAP: Mean arterial pressure
NOS: New Castle Ottawa
OR: Odds ratio
RCT: Randomized control trial
SBP: Systolic blood pressure
SCI: Spinal cord injury
SCPP: Spinal cord perfusion pressure
uOR: unadjusted Odds ratio
WHO: World Health Organization

## Declarations

FAZ currently has provisional patents (63/659, 989; provisional filed June 14, 2024 and 63/652,106; provisional filed May 27, 2024) and software copyrights (Canada IP Office Registration #: 1231355 and 1231080) held in relation to advanced brain monitoring devices and physiologic metric algorithms. All of this is unrelated to the content of this manuscript.

FAZ is supported through the Endowed MPI Chair in Neuroscience, the Natural Sciences and Engineering Research Council of Canada (NSERC; DGECR-2022-00260, RGPIN-2022-03621, ALLRP-578524-22, ALLRP-576386-22, I2IPJ 586104–23, ALLRP 586244-23, ALLRP-590680-2023, ALLRP 597442-24, ALLRP-597708-24, I2IPJ 2024-598125), the Canadian Institutes of Health Research (CIHR; Grant #: 530549), the MPI Neuroscience Research Operating Fund, the Health Sciences Centre Foundation Winnipeg, the Canada Foundation for Innovation (CFI) (Project #: 38583), Research Manitoba (Grant #s: 3906, 5429, 5914, 6554), and the Pan Am Clinic Foundation of Winnipeg.
