# Supplementary material for: Effect of blood pressure threshold on adverse outcomes in patients with acute spinal cord injury: a systematic review and meta-analysis
Source: Crit Care. 2025 Jul 16;29:308. doi: 10.1186/s13054-025-05531-3 (PMC12265288; doi:10.1186/s13054-025-05531-3)
Supplement: Supplementary file 1 — Additional file 1. Supplementary Appendix: Contains all supplementary information and data as referenced in manuscript. [file 13054_2025_5531_MOESM1_ESM.docx]

**Supplementary Appendix**

Tanvir A, Aziz Rizk A, Wang W, et al. Effect of Blood Pressure Threshold on Adverse Outcomes in Patients with Acute Spinal Cord Injury: A Systematic Review and Meta-Analysis.

[**eTable 1.**](#_eTable_1:_Search) Search Strategy

[**eTable 2.**](#_eTable_2:_Summary) Summary of Blood Pressure Management Strategies for Hypotension

[**eTable 3.**](#_eTable_3:_Adjusted) Adjusted Factors for Calculation of Adjusted Odds Ratios

[**eTable 4.**](#_eTable_4:_Quality) Quality Assessment Using Newcastle-Ottawa Scale for Cohort Studies

[**eTable 5.**](#_eTable_5:_Quality) Quality Assessment Using Cochrane Risk of Bias Assessment for RCTs

[**eTable 6.**](#_eTable_6:_Heterogeneity) Heterogeneity Analysis for Categorical Variables

[**eTable 7.**](#_eTable_7:_Leave) Leave-One-Out Analysis for Studies Reporting Crude OR

[**eTable 8.**](#_eTable_8:_Leave) Leave-One-Out Analysis for Studies Reporting Adjusted OR

[**eTable 9.**](#_eTable_9._Reasons) Reasons for Full-Text Exclusion

[**eTable 10.**](#_eTable_10._GRADE) GRADE classifications

[**eTable 11**](#_eTable_11._PRISMA)**.** PRISMA Checklist

[**eTable 12.**](#_eTable_12._MOOSE) MOOSE Checklist

[**eFigure 1.**](#_eFigure_1:_Forest) Forest plot showing crude association of hypotension on adverse functional outcome in patients with spinal cord injury

[**eFigure 2.**](#_eFigure_2:_Forest) Forest plot showing subgroup analysis by blood pressure type

[**eFigure 3.**](#_eFigure_3:_Forest) Forest plot showing subgroup analysis by mean arterial pressure threshold

[**eFigure 4.**](#_eFigure_4:_Forest) Forest plot showing subgroup analysis by ASIA grade injury

[**eFigure 5.**](#_eFigure_5:_Forest) Forest plot showing subgroup analysis by spinal level

[**eFigure 6.**](#_eFigure_6:_Forest) Forest plot showing subgroup analysis by trauma type (isolated SCI only vs isolated and polytrauma)

[**eFigure 7.**](#_eFigure_7:_Forest) Forest plot showing subgroup analysis by SCI etiology (traumatic vs non-traumatic)

[**eFigure 8.**](#_eFigure_8:_Forest) Forest plot showing subgroup analysis by functional outcome type

[**eFigure 9.**](#_eFigure_9:_Funnel) Funnel Plot for Publication Bias

[**eFigure 10.**](#_eFigure_10:_Galbraith) Galbraith Plot for Exhibiting Outliers

### **eTable 1:** Search Strategy

**MEDLINE**

**Ovid MEDLINE(R) 1946 to May 30, 2024**

| # | Searches |
| --- | --- |
| 1 | exp Spinal Injuries/ or exp Spinal Cord Injuries/ or exp Spinal Diseases/ or exp Spinal Cord Diseases/ or exp Spinal Fusion/ or exp Diskectomy/ or exp Laminectomy/ or exp Laminoplasty/ or exp Whiplash Injuries/ |
| 2 | ((spine or spinal or vertebra* or intervertebra*) adj3 (tumor* or tumour* or neoplas* or cancer* or sarcoma* or malignanc* or injur* or compress* or myelopath* or stenosis or stenoses or ischemi* or ischaemi* or surger* or fusion* or trauma* or fractur* or disease* or disorder* or deform* or malform* or dislocat* or patholog* or inflam* or myeliti* or degenerat* or laminectom* or laminoplast*)).ab,kf,ti. |
| 3 | ("diskectom*" or "discectom* laminectom* " or "laminoplast* " or "myeliti** " or "spondyliti*" or "spondylosis" or "spondylolysis" or "spondylodesis" or "spondylolisthesis" or "spondyloses" or "myelopath*" or "syringomyelia").ab,kf,ti. |
| 4 | 1 or 2 or 3 |
| 5 | exp Hypotension/ or exp Blood Pressure/ |
| 6 | ("hypotens* " or "blood pressure* " or "systolic pressure* " or "arterial pressure* " or "spinal cord perfusion pressure* ").ab,kf,ti. |
| 7 | 5 or 6 |
| 8 | 4 and 7 |
| 9 | exp "review"/ or exp meta analysis/ or case report/ or editorial/ or letter/ or (case report or systematic review or metaanalys* or meta-analys* or letter or comment or editorial).ti. |
| 10 | 8 not 9 |
| 11 | (exp child/ or pediatrics/ or exp infant/ or adolescent/) not adult/ |
| 12 | 10 not 11 |
| 13 | (exp animal/ or exp animal experiment/ or exp animal model/) not human/ |
| 14 | 12 not 13 |

### **eTable 2:** Summary of blood pressure management strategies for hypotension

| Study | Type of blood pressure | Hypotension threshold (mmHg) | Vasopressors  used | | Other BP treatments | | Complications related to treatment |
| --- | --- | --- | --- | --- | --- | --- | --- |
| Agarwal 2022 | MAP | 76 | Dopamine, Phenylephrine | | - | | Dopamine associated with higher rates of cardiovascular complications |
| Alfin 2023 | MAP | 80 | - | | - | | - |
| Balasuberamaniam 2023 | MAP | 80 | Epinephrine, Norepinephrine, Vasopressin, Phenylephrine, Dopamine | | - | | - |
| Blue 2022 | MAP | 65 | Names not specified | | Fluids | | - |
| Catapano 2016 | MAP | 85 | Dopamine, phenylephrine | | - | | Dopamine use was associated with a higher risk of complications, followed by phenylephrine |
| Chen 2022 | SBP | - | - | | - | | - |
| Chen 2017 | SCPP | - | Names not specified | | - | | Cardiac dysrhythmias associated with vasopressor use, particularly in older patients |
| Chiang 2022 | SBP | 80 | Ephedrine, Norepinephrine | | - | | Patients requiring vasopressors to manage intraoperative hypotension experienced a significantly increased risk of AKI |
| Cohn 2010 | MAP | Variable | Dopamine, Phenylephrine | | - | | - |
| Dakson 2017 | MAP | 85 | Dopamine, Norepinephrine | | Fluids | | - |
| Ehsanian 2020 | MAP | 50 | Phenylephrine, Ephedrine, Dopamine, Norepinephrine, Vasopressin | | - | | - |
| Gallagher 2020 | SCPP | - | - | | - | | - |
| Glassman 2024 | MAP | 65 | Norepinephrine | | CaCl_2_ | | Patients with no complications received less vasopressors than those with complications |
| Haldrup 2020 | MAP | 80 | Phenylephrine, Norepinephrine | | - | | - |
| Hawryluk 2015 | MAP | 85 | Phenylephrine, Norepinephrine, Dopamine | | - | | Vasopressor use was associated with cardiac dysrhythmias and ischemia |
| Hogg 2021^a^ | SCPP | 60 | Norepinephrine | | - | | - |
| Hogg 2021^b^ | SCPP | 60 | Norepinephrine | | - | | - |
| Jiang 2019 | MAP, SBP | 85, 90 | - | | - | | - |
| Kamel 2016 | MAP | 55 | - | | Fluids, Blood | | - |
| Långsjö 2024 | MAP | 85 | Norepinephrine | | Fluids, Blood | | - |
| LaRiccia 2023 | MAP | 85 | Norepinephrine | | - | | The most common vasopressor related complications were tachycardia and atrial fibrillation |
| Li 2019 | MAP | Variable | Phenylephrine | | - | | - |
| Martin 2015 | MAP | 85 | Names not specified | | - | | - |
| Müller 2023 | SBP | - | Norepinephrine | | Fluids | | - |
| Mushlin 2020 | MAP | 85 | - | | Fluids, Blood | | Study reported intolerance in some patients due to dysrhythmias and poor cardiac reserve. |
| Rask 2024 | MAP | 85 | Dobutamine, Norepinephrine | | Fluids, Blood | | - |
| Readdy 2016 | MAP | 85 | Dopamine, Phenylephrine | | - | | Dopamine was associated with higher complication rates (arrhythmias) compared to phenylephrine. |
| Rerikh 2020 | MAP | 85 | Norepinephrine, Dopamine, Dobutamine | | Methylpredn-isolone | | Complications were less frequent in the vasopressor group compared to the methylprednisolone group. |
| Santos 2014 | SBP | 90 | Norepinephrine, Dopamine | | - | | - |
| Sharma 2024 | MAP | - | - | | - | | - |
| Squair 2019 | MAP, SCPP | 80, 60 | Norepinephrine, Phenylephrine, Dopamine | | Fluids, Blood | | - |
| Squair 2017 | MAP, SCPP | 80, 50 | Norepinephrine, Phenylephrine, Dopamine | | Fluids, Blood | | - |
| Tee 2013 | SBP | 100 | - | | - | | - |
| Visagan 2023 | MAP | 85 | Norepinephrine | | - | | - |
| Vale 1997 | MAP | 85 | Dopamine, Norepinephrine | | Fluids, Colloids | | The study explicitly noted that there were no significant complications related to the use of vasopressors or aggressive blood pressure management |
| Weinberg 2020 | MAP | 85 | Norepinephrine, Phenylephrine | | - | | Vasopressor use was associated with an increased risk of in-hospital complications |
| Werndle 2024 | SCPP | 60 | Norepinephrine | | Metaraminol | | - |
| Zhang 2024 | MAP | 70 | - | | - | | - |
|  |  |  |  |  | |  |  |

Dashes represent missing data.

### **eTable 3:** Adjusted factors for calculation of adjusted odds ratios

| Study | Adjusted factors |
| --- | --- |
| Jiang 2019 | - Age, baseline AIS grade, injury level |
| Kamel 2016 | - Age, gender, ASA physical status, body mass index, history of chronic hypertension, diabetes mellitus, peripheral vascular disease, anemia, and intraoperative variables such as surgical procedure, anatomical location of the procedure, duration of surgery |
| Tee 2013 | - Age, injury severity score, blood sugar level, vital signs, traumatic brain injury, comorbidities, coagulation profile, neurology, and spine injury characteristics. |
| Weinberg 2020 | - Central cord syndrome, vasopressor dose, Injury Severity Score (ISS), admission ASIA grade, and the number of MAP recordings over the first 72 hours of admission |

### **eTable 4:** Quality assessment using Newcastle-Ottawa Scale for Cohort Studies

| Study | Representativeness | Selection Non-Exposed | Ascertainment of Exposure | Outcome Not Present at Start | Comparability | Additional Factor | Assessment of Outcome | Follow-Up Length | Adequacy of Follow Up | Total | Quality |
| --- | --- | --- | --- | --- | --- | --- | --- | --- | --- | --- | --- |
| Agarwal  2022 | Y | Y | Y | Y | Y | Y | Y | N | N | 7 | GOOD |
| Alfin  2023 | Y | Y | Y | Y | N | N | Y | N | N | 5 | FAIR |
| Balasuberamaniam 2023 | Y | Y | Y | Y | Y | N | Y | Y | Y | 8 | GOOD |
| Blue  2022 | Y | Y | Y | Y | Y | Y | Y | Y | Y | 9 | GOOD |
| Catapano  2016 | Y | Y | Y | Y | Y | N | Y | N | N | 6 | FAIR |
| Chen  2022 | Y | Y | Y | Y | Y | Y | Y | Y | Y | 9 | GOOD |
| Chen  2017 | Y | Y | Y | Y | Y | N | Y | Y | Y | 8 | GOOD |
| Chiang 2022 | Y | Y | Y | Y | Y | Y | Y | Y | Y | 9 | GOOD |
| Cohn 2010 | Y | Y | Y | Y | N | Y | Y | Y | Y | 8 | GOOD |
| Dakson  2017 | Y | Y | Y | Y | Y | N | Y | Y | Y | 8 | GOOD |
| Ehsanian 2020 | N | Y | Y | Y | N | N | Y | Y | Y | 6 | FAIR |
| Gallagher 2020 | Y | Y | Y | Y | Y | N | Y | Y | Y | 8 | GOOD |
| Glassman  2024 | Y | Y | Y | Y | Y | Y | Y | Y | Y | 9 | GOOD |
| Haldrup  2020 | Y | Y | Y | Y | N | N | Y | Y | Y | 7 | GOOD |
| Hawryluk  2015 | Y | Y | Y | Y | N | N | Y | Y | Y | 7 | GOOD |
| Hogg 2021a | Y | Y | Y | Y | Y | Y | Y | Y | Y | 9 | GOOD |
| Hogg  2021b | Y | Y | Y | Y | Y | N | Y | Y | Y | 8 | GOOD |
| Jiang  2019 | Y | Y | Y | Y | Y | Y | Y | Y | Y | 9 | GOOD |
| Kamel 2016 | Y | Y | Y | Y | Y | Y | Y | N | N | 7 | GOOD |
| Långsjö 2024 | Y | Y | Y | Y | Y | N | Y | Y | Y | 8 | GOOD |
| LaRiccia 2023 | Y | Y | Y | Y | Y | N | Y | Y | Y | 8 | GOOD |
| Li  2019 | Y | Y | Y | Y | N | N | Y | Y | Y | 7 | GOOD |
| Martin  2015 | Y | Y | Y | Y | Y | N | Y | Y | Y | 8 | GOOD |
| Müller  2023 | N | Y | Y | Y | Y | Y | Y | Y | N | 7 | GOOD |
| Mushlin  2020 | Y | Y | Y | Y | Y | Y | Y | Y | Y | 9 | GOOD |
| Rask 2024 | Y | Y | Y | Y | Y | Y | Y | Y | Y | 9 | GOOD |
| Readdy  2016 | Y | Y | Y | Y | Y | N | Y | Y | Y | 8 | GOOD |
| Rerikh  2020 | Y | Y | Y | Y | Y | N | Y | Y | Y | 8 | GOOD |
| Santos  2014 | Y | Y | Y | Y | Y | Y | Y | Y | Y | 9 | GOOD |
| Squair  2019 | Y | Y | Y | Y | Y | N | Y | Y | Y | 8 | GOOD |
| Squair  2017 | Y | Y | Y | Y | Y | Y | Y | Y | Y | 9 | GOOD |
| Tee  2013 | Y | Y | Y | Y | Y | Y | Y | Y | Y | 9 | GOOD |
| Visagan  2023 | Y | Y | Y | Y | Y | Y | Y | Y | Y | 9 | GOOD |
| Vale  1997 | N | Y | Y | Y | N | N | Y | Y | Y | 6 | FAIR |
| Weinberg  2020 | Y | Y | Y | Y | Y | N | Y | Y | Y | 8 | GOOD |
| Werndle  2024 | Y | Y | Y | Y | Y | Y | Y | Y | Y | 9 | GOOD |
| Zhang  2024 | Y | Y | Y | Y | Y | N | Y | Y | Y | 8 | GOOD |

### **eTable 5:** Quality assessment using Cochrane Risk of Bias Assessment for RCTs

| Study | Random sequence generation? (selection bias) | Allocation concealment? (selection bias) | Blinding of participants, personnel, and outcome assessors? (performance and detection bias) | Incomplete outcome data? (attrition bias) | Selective reporting? (reporting bias) | Other bias? | Overall risk of bias |
| --- | --- | --- | --- | --- | --- | --- | --- |
| Sharma 2024 | Y | Y | N | N | N | N | LOW |

### **eTable 6:** Heterogeneity analysis for categorical variables

| **Group** | **tau2** | **I2** |
| --- | --- | --- |
| BP Type   - MAP - SBP - SCPP | 0.501  0.018  0.000 | 70.20  10.86  0.11 |
| MAP Threshold   - <85mmHg - ≥85mmHg | 0.59  0.00 | 70.88  0.00 |
| Functional Outcome   - Neuromotor recovery - Visceral function | 0.540  0.060 | 70.22  29.84 |
| SCI Etiology   - Traumatic - Non-Traumatic | 0.527  0.000 | 72.96  0.00 |
| Trauma Type   - Isolated only - Isolated and Polytrauma | 0.404  0.705 | 70.20  66.31 |
| Spinal Level   - Cervical - Thoracolumber - Cervical, Thoracic, Lumber | 0.000  0.000  0.751 | 0.00  0.00  80.43 |
| Motor Complete Category   - <50% Motor Complete SCI - ≥50% Motor Complete SCI | 0.000  0.817 | 0.00  64.50 |

### **eTable 7:** Leave one out analysis for studies reporting crude OR (29 studies)


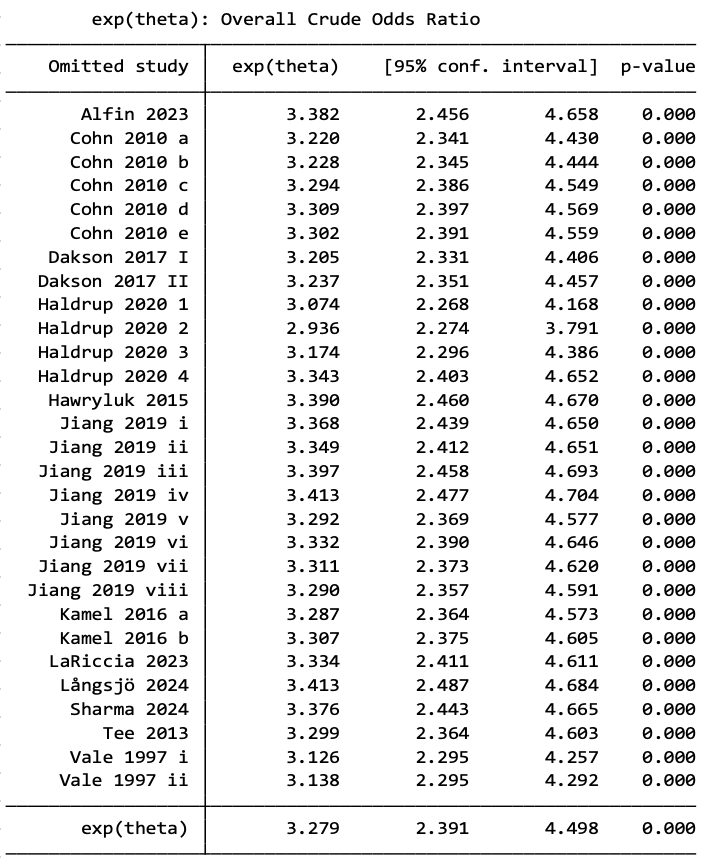


Cohn 2010 sub-studies include different MAP thresholds of a) 65mmHg, b) 70mmHg, c) 75 mmHg, d) 80mmHg, e) 85mmHg. Dakson 2017 sub-studies include different spinal levels of I) all spinal levels, II) cervical levels only. Haldrup 2020 sub-studies include different location of hypotension of 1) prehospital MAP, 2) intraoperative MAP, 3) NICU day 1-2 MAP, 4) NICU day 3-7 MAP. Jiang 2019 sub-studies include different outcomes and blood pressure type of i) association of SBP and resp function, ii) association of SBP and ambulation, iii) association of SBP and bladder function, iv) SBP and bowel function, v) association of MAP and resp function, vi) association of MAP and ambulation, vii) association of MAP and bladder function, viii) association of MAP and bowel function. Kamel 2016 sub-studies include different MAP thresholds of a) 55mmHg, b) 80mmHg. Vale 1997 sub-studies include different outcomes of a) association of MAP and no ambulatory capacity, b) association of MAP and bladder/bowel function.

### **eTable 8:** Leave one out analysis for studies reporting adjusted OR (16 studies)


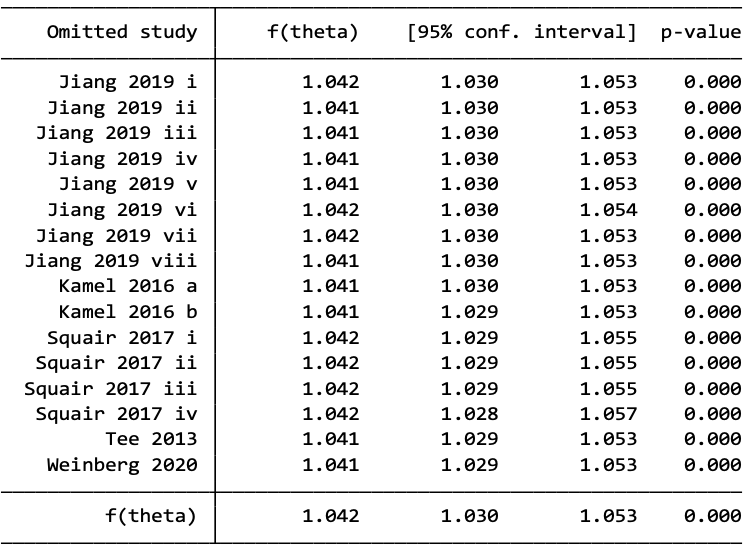


Jiang 2019 sub-studies include different outcomes and blood pressure type of i) association of SBP and resp function, ii) association of SBP and ambulation, iii) association of SBP and bladder function, iv) association of SBP and bowel function, v) association of MAP and resp function, vi) association of MAP and ambulation, vii) association of MAP and bladder function, viii) association of MAP and bowel function. Kamel 2016 sub-studies include different MAP thresholds of a) 55mmHg, b) 80mmHg. Squair 2017 sub-studies include different outcomes and blood pressure type of i) association of MAP and change in AIS grade (conversion), ii) association of MAP and change in motor score, iii) association of SCPP and change in AIS grade (conversion, iv) association of SCPP and motor score.

###

### **eTable 9.** Reasons for Full-Text Exclusion

|  | Title | First Author | Year Published | Reasons for Exclusion |
| --- | --- | --- | --- | --- |
| 1 | Leveraging Machine Learning to Identify an Intraoperative Mean Arterial Pressure Threshold for Neurological Improvement in Patients Undergoing Decompressive Surgery for Acute Spinal Cord Injury | Aabedi | 2022 | Full text not found |
| 2 | Postural effects on blood pressure, intraocular pressure, and ocular perfusion pressure in patients with spinal cord injury | Abd Manan | 2024 | chronic SCI, not acute |
| 3 | Enhanced Intraoperative Fluid Resuscitation Guidelines to Reduce Postoperative Length of Stay | Akinfe | 2023 | Ineligible publication type |
| 4 | Medical Communication Services after Traumatic Spinal Cord Injury | Alshorman | 2021 | Ineligible publication type |
| 5 | Blood pressure management in acute spinal cord injury pre-and post-decompression surgery | Altaf | 2014 | Ineligible publication type |
| 6 | Respiratory and cardiovascular modulations in patients with spinal cord injury | Aslan | 2015 | Full text not found |
| 7 | Ambulatory blood pressure monitoring in patients with spinocerebellar degeneration. | Azuma | 2002 | Not acute SCI |
| 8 | Haemodynamic management in early spinal cord injury care: Findings from a prospective study and clinician survey | Ball | 2018 | Ineligible publication type |
| 9 | The effect of nitric oxide inhibition in spinal cord injured humans with and without preserved sympathetic control of the vasculature | Brown | 2016 | Not acute SCI |
| 10 | Assessing the integrity of sympathetic pathways in spinal cord injury | Brown | 2007 | Wrong intervention |
| 11 | Outcome of Surgical and Intensive Care Treatment of Patients with Traumatic Cervical Spinal Cord Injury: A Single Center, Cross-Sectional, Retrospective Study | Catkin | 2023 | Wrong intervention |
| 12 | The incidence of vision loss due to perioperative ischemic optic neuropathy associated with spine surgery - The Johns Hopkins hospital experience | Chang | 2005 | Ineligible publication type |
| 13 | Anesthesia and Perioperative Care in Cervical Spinal Surgery | Chierichini | 2022 | Ineligible publication type |
| 14 | Prehospital Cardiovascular Autoregulatory Disturbances Correlate With the Functional Neuroanatomy of Acute Spinal Cord Injury | Clark | 2023 | No outcomes |
| 15 | Intra-operative mean arterial blood pressure predicts neurologic recovery following acute traumatic spinal cord injury | Cook | 2021 | Full text not found |
| 16 | Optimization of the medical and surgical management of traumatic spinal cord injury | Dakson | 2016 | Ineligible publication type |
| 17 | Investigating the implementation of acute care guidelines for spinal cord injuries in Nova Scotia from 2005-2010 | Dakson | 2016 | Full text not found |
| 18 | Altered left ventricular diastolic function in subjects with spinal cord injury | DeRossi | 2016 | Ineligible publication type |
| 19 | Exploration of surgical blood pressure management and expected motor recovery in individuals with traumatic spinal cord injury | Ehsanian | 2020 | Duplicate |
| 20 | Cardiovascular stress during inpatient spinal cord injury physical and occupational therapy | Eng | 2015 | Ineligible publication type |
| 21 | Acute Treatment of Spinal Cord Injury. | Esce | 2000 | Ineligible publication type |
| 22 | Treating acute spinal cord patients with a spinal cord perfusion pressure management protocol | Fields | 2022 | Ineligible publication type |
| 23 | Perioperative and intraoperative predictors of ICU length of stay in patients undergoing long fusion spinal surgery | Gazendam | 2017 | Ineligible publication type |
| 24 | All over the MAP: describing pressure variability in acute spinal cord injury | Gee | 2022 | No outcomes |
| 25 | Blood pressure in spinal cord injury | Gotshall | 1971 | Full text not found |
| 26 | Assessment of non-neurosurgical complications in traumatic neurosurgical patients admitted in ICU: A prospective observational study | Goyal | 2018 | Ineligible publication type |
| 27 | Blood pressure management after acute spinal cord injury | Hadley | 2002 | Ineligible publication type |
| 28 | Traumatic spinal cord injury and 24-hour blood pressure profile during primary rehabilitation | Hagen | 2015 | Full text not found |
| 29 | Acute kidney injury in elderly patients undergoing spinal surgery | Harrison | 2018 | Full text not found |
| 30 | The cause of neurologic deterioration after acute cervical spinal cord injury | Harrop | 2001 | Wrong intervention |
| 31 | Higher mean arterial blood pressures following human spinal cord injury correlate with greater neurological recovery | Hawryluk | 2014 | Duplicate |
| 32 | Adverse effects of vasopressor support for the maintenance of mean arterial pressure in acute spinal cord injuries | Hiatt | 2017 | Ineligible publication type |
| 33 | Mean arterial pressure maintenance following spinal cord injury: does meeting the target matter? | Hinckley | 2021 | Full text not found |
| 34 | Characteristics of cardiovascular responses to an orthostatic challenge in trained spinal cord-injured individuals | Itoh | 2018 | Wrong intervention |
| 35 | Intact sympathetic nervous system is required for leptin effects on resting metabolic rate in people with spinal cord injury | Jeon | 2003 | Wrong intervention |
| 36 | Postoperative visual changes following spine surgery | Karakoc | 2012 | Wrong outcomes |
| 37 | Blood pressure instability in persons with SCI: Evidence from a 30-day home monitoring observation | Katzelnick | 2019 | Not acute SCI |
| 38 | A study to evaluate ocular changes in patients undergoing spine surgery in the prone position | Kaur | 2021 | Wrong outcomes |
| 39 | The Effect of Fluid Management & Intraoperative Blood Pressure on Patients' Outcome following Complex Spine Surgeries | Koh | 2023 | Ineligible publication type |
| 40 | A prospective evaluation of hemodynamic management in acute spinal cord injury patients | Kong | 2013 | Wrong outcomes |
| 41 | Spinal cord injury: epidemiological study of 386 cases with emphasis on those patients admitted more than four hours after the trauma. | Leal-Filho | 2008 | Wrong intervention |
| 42 | Cardiovascular-abnormalities accompanying acute spinal-cord injury in humans - incidence, time course and severity | Lehmann | 1987 | Wrong outcomes |
| 43 | Under Pressure: For new patients with paralysis, tighter blood pressure control management may lead to more mobility. | Leigh | 2022 | Ineligible publication type |
| 44 | Hemodynamic parameters in patients with acute cervical cord trauma: Description, intervention, and prediction of outcome | Levi | 1993 | Wrong intervention |
| 45 | The acutely injured cord and vasopressors: Proceed with caution in the frail | Malcolm | 2020 | Ineligible publication type |
| 46 | Effect of Multiple Trauma on Mortality and Neurological Recovery after Spinal Cord or Cauda Equina Injury | Meguro | 1988 | Wrong intervention |
| 47 | Evaluating optimal vasopressor use in neurogenic shock | Miller | 2011 | Ineligible publication type |
| 48 | An investigation to the prolonged requirement (>7 days) of vasopressors in cervical spinal cord injury patients-a retrospective analysis | Mishra | 2021 | Wrong intervention |
| 49 | Vasopressor choice in prone spine surgery: An examination of intraoperative blood pressure control in the elderly. A retrospective study | Nair | 2012 | Ineligible publication type |
| 50 | Long-Term Outcome of Acute Spinal Cord Ischemia Syndrome | Nedeltchev | 2004 | Wrong intervention |
| 51 | Spinal cord ischemia: Clinical and imaging patterns, pathogenesis, and outcomes in 27 patients | Novy | 2006 | Wrong intervention |
| 52 | Early management of mean arterial pressure affects recovery after spinal cord injury | Pan | 2018 | Full text not found |
| 53 | Monitoring intraspinal and spinal cord perfusion pressure in acute spinal cord injury | Papadopoulos | 2015 | Ineligible publication type |
| 54 | Prehospital hemodynamic status and neurologic outcomes following acute traumatic spinal cord injury | Patterson | 2021 | Full text not found |
| 55 | Regional cerebral blood flow responses to rapid reductions in blood pressure after high level spinal cord injury: the effect of alpha1-agonist | Phillips | 2014 | Ineligible publication type |
| 56 | A national sample of variation in blood pressure and anemia severity in spinal fusion surgery | Posner | 2009 | Ineligible publication type |
| 57 | Heart rate variability predicts post-induction hypotension in patients with cervical myelopathy | Raghavan | 2019 | Wrong outcomes |
| 58 | Effect of continuous propofol infusion and hypotension on somatosensory evoked-potentials in 9 patients under-going posterior spinal-fusion | Restaflarer | 1993 | Full text not found |
| 59 | Dose response effect of exposure to hypotension on expected neurological recovery in individuals with traumatic spinal cord injury | Reza | 2016 | Ineligible publication type |
| 60 | Effects of compression stockings on sympathetic activity and heart rate variability in individuals with spinal cord injury | Rimaud | 2012 | Wrong intervention |
| 61 | Effects of propofol or sevoflurane anesthesia induction on hemodynamics in patients undergoing fiberoptic intubation for cervical spine surgery: A randomized, controlled, clinical trial | Robba | 2017 | Wrong outcomes |
| 62 | Induced hypertension does not improve out-comes in penetrating spinal cord injury | Saigal | 2014 | Full text not found |
| 63 | Comparison of the effects of arterial blood pressure and cardiac output based hemodynamic management on cognitive function in elderly patients undergoing spinal surgery: a Randomized Clinical Trial | Saka | 2021 | Wrong intervention |
| 64 | A Hemodynamic Safety Checklist Can Improve Blood Pressure Monitoring in Patients with Acute Spinal Cord Injury | Sewell | 2019 | Wrong outcomes |
| 65 | Motor and autonomic concomitant health improvements with neuromodulation and exercise (MACHINE) training: a randomised controlled trial in individuals with spinal cord injury | Shackleton | 2023 | Not acute SCI |
| 66 | Hypotensive episodes early after sci associated with lower map in ICU: A prospective track-sci study | Singh | 2018 | Ineligible publication type |
| 67 | Cardiovascular status of individuals with incomplete spinal cord injury from 7 neurorecovery network rehabilitation centers | Sisto | 2012 | Wrong outcomes |
| 68 | Early Predictors of Functional Disability After Spine Trauma A Level 1 Trauma Center Study | Tee | 2013 | Duplicate |
| 69 | Hemodynamic parameters and timing of surgical decompression in acute cervical spinal cord injury | Tuli | 2007 | Ineligible population |
| 70 | Vasopressor Outcomes in Spine Surgery | Unknown | 2023 | Full text not found |
| 71 | HPI Algorithm for the Prevention of IOH During Spinal Surgery | Unknown | 2022 | Full text not found |
| 72 | Effect of individualized blood pressure control on troponin and renal function in elderly patients undergoing spinal surgery | Unknown | 2020 | Full text not found |
| 73 | Correlation Analysis of the Effects of Management Pressure Goals and Control Volume Goals in Patients in Spinal Surgery | Unknown | 2019 | Full text not found |
| 74 | Mean Arterial Blood Pressure Treatment for Acute Spinal Cord Injury | Unknown | 2014 | Full text not found |
| 75 | Arterial Pulse Waveform Contour Analysis for Intraoperative Goal Directed Therapy in Major Spine Surgery | Unknown | 2010 | Full text not found |
| 76 | Abdominal binder effect on respiratory, voice, and haemodynamic outcomes after a tetraplegic spinal cord injury | Wadsworth | 2011 | Ineligible publication type |
| 77 | Changes in intraocular pressure due to surgical positioning: Studying potential risk for postoperative vision loss | Walick | 2007 | Wrong outcomes |
| 78 | Double-blinded, placebo-controlled crossover trial to determine the effects of midodrine on blood pressure during cognitive testing in persons with SCI | Wecht | 2020 | Not acute SCI |
| 79 | Evaluation of Cardiovascular Autonomic Function during Inpatient Rehabilitation following Traumatic Spinal Cord Injury | Wecht | 2022 | Wrong intervention |
| 80 | Measurement and optimisation of spinal cord perfusion pressure in acute spinal cord injury | Werndle | 2013 | Ineligible publication type |
| 81 | Measurement and optimisation of spinal cord perfusion pressure in acute spinal cord injury | Werndle | 2014 | Duplicate |
| 82 | Neurogenic shock treatment effectiveness in the emergency department-the need for a code sci | Whetstone | 2019 | Ineligible publication type |
| 83 | Norepinephrine in Goal-Directed Fluid Therapy During General Anesthesia in Elderly Patients Undergoing Spinal Operation: Determining Effective Infusion Rate to Enhance Postoperative Functions | Wu | 2021 | No outcomes |
| 84 | Importance of checking prehospital neurological findings to reveal incidence of spinal cord concussion | Yanagawa | 2012 | Wrong intervention |
| 85 | Acute Effects of Whole Body Vibration on Central and Peripheral Hemodynamics and Oxygen Consumption in Individuals with Spinal Cord Injury. | Yarar | 2011 | Wrong intervention |
| 86 | Anaesthetic requirements and stress hormone responses in acute cord-injured patients undergoing surgery at the injured spine | Yoo | 2009 | Wrong outcomes |
| 87 | Hemodynamic effects of withholding vs. continuing angiotensin II receptor blockers on the day of prone positioning spinal surgery in elderly patients | Yuan | 2021 | Wrong outcomes |
| 88 | Study on relationship between blood pressure and adverse symptoms of autonomic nerve reflex in measurement of bladder pressure in patients with spinal cord injury. | Yuan Lixiu | 2015 | Full text not found |
| 89 | Clinical Implementation of Novel Spinal Cord Perfusion Pressure Protocol in Acute Traumatic Spinal Cord Injury at U.S. Level I Trauma Center: TRACK-SCI Study | Yue | 2020 | Wrong intervention |
| 90 | Initial Experience of Spinal Cord Perfusion Pressure Goals in Lieu of Mean Arterial Pressure Goals in Acute Traumatic Spinal Cord Injury at a United States Level I Trauma Center: A Transforming Research and Clinical Knowledge-Spinal Cord Injury Study | Yue | 2019 | Ineligible publication type |
| 91 | Hemodynamic responses to penetrating spinal cord injuries | Zipnick | 1993 | Wrong outcomes |
| 92 | [Cognitive status assessment after spinal surgery]. | Unknown | 2012 | Full text not found |

### **eTable 10.** GRADE classifications

| **Main Outcomes** | **No. of participants (studies)** | **Study limitations** | **Consistency** | **Directness** | **Precision** | **Publication bias** | **Relative effect (95% CI)** | **Quality of the evidence (GRADE)** |
| --- | --- | --- | --- | --- | --- | --- | --- | --- |
| **Lower blood pressure threshold on adverse outcomes** | 2,553 | Serious limitations (−1) | No important inconsistency | Direct | No important imprecision | Unlikely | uOR: 3.28 (95% CI: 2.39-4.50) | Moderate |
| **Subgroup analysis by blood pressure threshold type MAP on adverse outcomes** | 1,485 | Serious limitations (−1) | No important inconsistency | Direct | Imprecision (−1) | Unlikely | uOR: 3.83 (95% CI: 2.65-5.54) | Low |
| **Subgroup analysis by blood pressure threshold type SBP on adverse outcomes** | 1,368 | Serious limitations (−1) | No important inconsistency | Direct | No important imprecision | Unlikely | uOR: 2.00 (95% CI: 1.41-2.84) | Moderate |
| **Effects of lower blood pressure threshold based on cervical injuries on adverse outcomes** | 722 | Serious limitations (−1) | No important inconsistency | Direct | Imprecision (−1) | Unlikely | uOR: 2.71 (95% CI: 2.13-3.47) | Low |
| **Effects of lower blood pressure threshold based on thoracolumbar injuries on adverse outcomes** | 80 | Serious limitations (−1) | No important inconsistency | Direct | Imprecision (−1) | Unlikely | uOR: 3.00 (95% CI: 1.72-5.22) | Low |
| **Blood pressure thresholds and adverse functional outcomes based on severity of SCI: complete motor complete injury (ASIA A and B) in ≥ 50% of patients** | 433 | Serious limitations (−1) | No important inconsistency | Direct | Imprecision (−1) | Unlikely | uOR: 7.27 (95% CI: 2.35-22.48) | Low |
| **Blood pressure thresholds and adverse functional outcomes based on severity of SCI: complete motor complete injury (ASIA A and B) in <50% of patients** | 1,110 | Serious limitations (−1) | No important inconsistency | Direct | No important imprecision | Unlikely | uOR: 2.01 (95% CI: 1.18-3.43) | Moderate |
| **Subgroup analysis for studies that only reported isolated SCI** | 1,379 | Serious limitations (−1) | No important inconsistency | Direct | Imprecision (−1) | Unlikely | uOR: 3.15 (95% CI: 2.23-4.46) | Low |
| **Subgroup analysis for studies that reported both isolated and polytraumatic SCI** | 946 | Serious limitations (−1) | No important inconsistency | Direct | Imprecision (−1) | Unlikely | uOR: 3.89 (95% CI: 1.83–8.26) | Low |
| **Subgroup analysis based on SCI etiology for traumatic injury** | 2,113 | Serious limitations (−1) | No important inconsistency | Direct | Imprecision (−1) | Unlikely | uOR: 3.43 (95% CI: 2.40-4.89) | Low |
| **Subgroup analysis based on SCI etiology for non-traumatic injury** | 212 | Serious limitations (−1) | No important inconsistency | Direct | Imprecision (−1) | Unlikely | uOR: 2.57 (95% CI: 1.58-4.19) | Low |
| **Subgroup analysis to examine the impact of suboptimal blood pressure on neuromotor recovery** | 2,325 | Serious limitations (−1) | No important inconsistency | Direct | Imprecision (−1) | Unlikely | uOR: 3.61 (95% CI: 2.43-5.38) | Low |
| **Subgroup analysis to examine the impact of suboptimal blood pressure on visceral function** | 862 | Serious limitations (−1) | No important inconsistency | Direct | No important imprecision | Unlikely | uOR: 2.54 (95% CI: 1.81-3.56) | Moderate |

### **eTable 11.** PRISMA Checklist

| **Section and Topic** | **Item #** | **Checklist item** | **Location where item is reported** |
| --- | --- | --- | --- |
| **TITLE** | | | 1 |
| Title | 1 | Identify the report as a systematic review. | 1 |
| **ABSTRACT** | | | 2 |
| Abstract | 2 | See the PRISMA 2020 for Abstracts checklist. | 2 |
| **INTRODUCTION** | | | 5 |
| Rationale | 3 | Describe the rationale for the review in the context of existing knowledge. | 5 |
| Objectives | 4 | Provide an explicit statement of the objective(s) or question(s) the review addresses. | 5 |
| **METHODS** | | | 5-8 |
| Eligibility criteria | 5 | Specify the inclusion and exclusion criteria for the review and how studies were grouped for the syntheses. | 6 |
| Information sources | 6 | Specify all databases, registers, websites, organisations, reference lists and other sources searched or consulted to identify studies. Specify the date when each source was last searched or consulted. | 5-6 |
| Search strategy | 7 | Present the full search strategies for all databases, registers and websites, including any filters and limits used. | 5-6 |
| Selection process | 8 | Specify the methods used to decide whether a study met the inclusion criteria of the review, including how many reviewers screened each record and each report retrieved, whether they worked independently, and if applicable, details of automation tools used in the process. | 6-7 |
| Data collection process | 9 | Specify the methods used to collect data from reports, including how many reviewers collected data from each report, whether they worked independently, any processes for obtaining or confirming data from study investigators, and if applicable, details of automation tools used in the process. | 7-8 |
| Data items | 10a | List and define all outcomes for which data were sought. Specify whether all results that were compatible with each outcome domain in each study were sought (e.g. for all measures, time points, analyses), and if not, the methods used to decide which results to collect. | 7-8 |
|  | 10b | List and define all other variables for which data were sought (e.g. participant and intervention characteristics, funding sources). Describe any assumptions made about any missing or unclear information. | 7-8 |
| Study risk of bias assessment | 11 | Specify the methods used to assess risk of bias in the included studies, including details of the tool(s) used, how many reviewers assessed each study and whether they worked independently, and if applicable, details of automation tools used in the process. | 7-8 |
| Effect measures | 12 | Specify for each outcome the effect measure(s) (e.g. risk ratio, mean difference) used in the synthesis or presentation of results. | 7-8 |
| Synthesis methods | 13a | Describe the processes used to decide which studies were eligible for each synthesis (e.g. tabulating the study intervention characteristics and comparing against the planned groups for each synthesis (item #5)). | 7-8 |
|  | 13b | Describe any methods required to prepare the data for presentation or synthesis, such as handling of missing summary statistics, or data conversions. | 7 |
|  | 13c | Describe any methods used to tabulate or visually display results of individual studies and syntheses. | 7-8 |
|  | 13d | Describe any methods used to synthesize results and provide a rationale for the choice(s). If meta-analysis was performed, describe the model(s), method(s) to identify the presence and extent of statistical heterogeneity, and software package(s) used. | 6-8 |
|  | 13e | Describe any methods used to explore possible causes of heterogeneity among study results (e.g. subgroup analysis, meta-regression). | 8 |
|  | 13f | Describe any sensitivity analyses conducted to assess robustness of the synthesized results. | 7-8 |
| Reporting bias assessment | 14 | Describe any methods used to assess risk of bias due to missing results in a synthesis (arising from reporting biases). | 7 |
| Certainty assessment | 15 | Describe any methods used to assess certainty (or confidence) in the body of evidence for an outcome. | 7-8 |

| **Section and Topic** | **Item #** | **Checklist item** | **Location where item is reported** |
| --- | --- | --- | --- |
| **RESULTS** | |  | 8-18 |
| Study selection | 16a | Describe the results of the search and selection process, from the number of records identified in the search to the number of studies included in the review, ideally using a flow diagram. | 9 |
|  | 16b | Cite studies that might appear to meet the inclusion criteria, but which were excluded, and explain why they were excluded. | 9-10 |
| Study characteristics | 17 | Cite each included study and present its characteristics. | 10 |
| Risk of bias in studies | 18 | Present assessments of risk of bias for each included study. | 17 |
| Results of individual studies | 19 | For all outcomes, present, for each study: (a) summary statistics for each group (where appropriate) and (b) an effect estimate and its precision (e.g. confidence/credible interval), ideally using structured tables or plots. | 9-16 |
| Results of syntheses | 20a | For each synthesis, briefly summarise the characteristics and risk of bias among contributing studies. | 15-17 |
|  | 20b | Present results of all statistical syntheses conducted. If meta-analysis was done, present for each the summary estimate and its precision (e.g.  confidence/credible interval) and measures of statistical heterogeneity. If comparing groups, describe the direction of the effect. | 10-17 |
|  | 20c | Present results of all investigations of possible causes of heterogeneity among study results. | 15-17 |
|  | 20d | Present results of all sensitivity analyses conducted to assess the robustness of the synthesized results. | 16-17 |
| Reporting biases | 21 | Present assessments of risk of bias due to missing results (arising from reporting biases) for each synthesis assessed. | 16-17 |
| Certainty of evidence | 22 | Present assessments of certainty (or confidence) in the body of evidence for each outcome assessed. | 11-16 |
| **DISCUSSION** | |  | 17-24 |
| Discussion | 23a | Provide a general interpretation of the results in the context of other evidence. | 17-21 |
|  | 23b | Discuss any limitations of the evidence included in the review. | 18-24 |
|  | 23c | Discuss any limitations of the review processes used. | 18-22 |
|  | 23d | Discuss implications of the results for practice, policy, and future research. | 17-21 |
| **OTHER INFORMATION** | |  | 2-3, 24-25 |
| Registration and protocol | 24a | Provide registration information for the review, including register name and registration number, or state that the review was not registered. | 2 |
|  | 24b | Indicate where the review protocol can be accessed, or state that a protocol was not prepared. | 2 |
|  | 24c | Describe and explain any amendments to information provided at registration or in the protocol. | 2 |
| Support | 25 | Describe sources of financial or non-financial support for the review, and the role of the funders or sponsors in the review. | 3 |
| Competing  interests | 26 | Declare any competing interests of review authors. | 24-25 |
| Availability of data, code and other materials | 27 | Report which of the following are publicly available and where they can be found: template data collection forms; data extracted from included studies; data used for all analyses; analytic code; any other materials used in the review. | 2, 25 |

### **eTable 12.** MOOSE Checklist

| **Item No** | **Recommendation** | **Reported on Page No** |
| --- | --- | --- |
| Reporting of background should include | | |
| 1 | Problem definition | 5 |
| 2 | Hypothesis statement | 5 |
| 3 | Description of study outcome(s) | 5 |
| 4 | Type of exposure or intervention used | 5 |
| 5 | Type of study designs used | 5 |
| 6 | Study population | 5 |
| Reporting of search strategy should include | | |
| 7 | Qualifications of searchers (eg, librarians and investigators) | 6 |
| 8 | Search strategy, including time period included in the synthesis and key words | 5-6 |
| 9 | Effort to include all available studies, including contact with authors | 5-6 |
| 10 | Databases and registries searched | 6 |
| 11 | Search software used, name and version, including special features used (eg, explosion) | 7 |
| 12 | Use of hand searching (eg, reference lists of obtained articles) | 8 |
| 13 | List of citations located and those excluded, including justification | 7-8 |
| 14 | Method of addressing articles published in languages other than English | 7 |
| 15 | Method of handling abstracts and unpublished studies | 7 |
| 16 | Description of any contact with authors | 7 |
| Reporting of methods should include | | |
| 17 | Description of relevance or appropriateness of studies assembled for assessing the hypothesis to be tested | 8 |
| 18 | Rationale for the selection and coding of data (eg, sound clinical principles or convenience) | 7 |
| 19 | Documentation of how data were classified and coded (eg, multiple raters, blinding and interrater reliability) | 7 |
| 20 | Assessment of confounding (eg, comparability of cases and controls in studies where appropriate) | 7-8 |
| 21 | Assessment of study quality, including blinding of quality assessors, stratification or regression on possible predictors of study results | 7 |
| 22 | Assessment of heterogeneity | 8 |
| 23 | Description of statistical methods (eg, complete description of fixed or random effects models, justification of whether the chosen models account for predictors of study results, dose-response models, or cumulative meta-analysis) in sufficient detail to be replicated | 7-8 |
| 24 | Provision of appropriate tables and graphics | 7-8 |
| Reporting of results should include | | |
| 25 | Graphic summarizing individual study estimates and overall estimate | 14 |
| 26 | Table giving descriptive information for each study included | 11 |
| 27 | Results of sensitivity testing (eg, subgroup analysis) | 16-17 |
| 28 | Indication of statistical uncertainty of findings | 16-17 |

| **Item No** | **Recommendation** | **Reported on Page No** |
| --- | --- | --- |
| Reporting of discussion should include | | |
| 29 | Quantitative assessment of bias (eg, publication bias) | 17-18 |
| 30 | Justification for exclusion (eg, exclusion of non-English language citations) | 9 |
| 31 | Assessment of quality of included studies | 17-18 |
| Reporting of conclusions should include | | |
| 32 | Consideration of alternative explanations for observed results | 21-23 |
| 33 | Generalization of the conclusions (ie, appropriate for the data presented and within the domain of the literature review) | 22-24 |
| 34 | Guidelines for future research | 23-24 |
| 35 | Disclosure of funding source | 3 |

### **eFigure 1:** Forest plot showing crude association of hypotension on adverse functional outcome in patients with spinal cord injury (Effect estimate = unadjusted odds ratio) (29 studies)

Legend: Cohn 2010 sub-studies include different MAP thresholds of a) 65mmHg, b) 70mmHg, c) 75 mmHg, d) 80mmHg, e) 85mmHg. Dakson 2017 sub-studies include different spinal levels of I) all spinal levels, II) cervical levels only. Haldrup 2020 sub-studies include different location of hypotension of 1) prehospital MAP, 2) intraoperative MAP, 3) NICU day 1-2 MAP, 4) NICU day 3-7 MAP. Jiang 2019 sub-studies include different outcomes and blood pressure type of i) association of SBP and resp function, ii) association of SBP and ambulation, iii) association of SBP and bladder function, iv) SBP and bowel function, v) association of MAP and resp function, vi) association of MAP and ambulation, vii) association of MAP and bladder function, viii) association of MAP and bowel function. Kamel 2016 sub-studies include different MAP thresholds of a) 55mmHg, b) 80mmHg. Vale 1997 sub-studies include different outcomes of a) association of MAP and no ambulatory capacity, b) association of MAP and bladder/bowel function.

### **eFigure 2:** Forest plot showing subgroup analysis by blood pressure type (Effect estimate = unadjusted odds ratio) (29 studies)


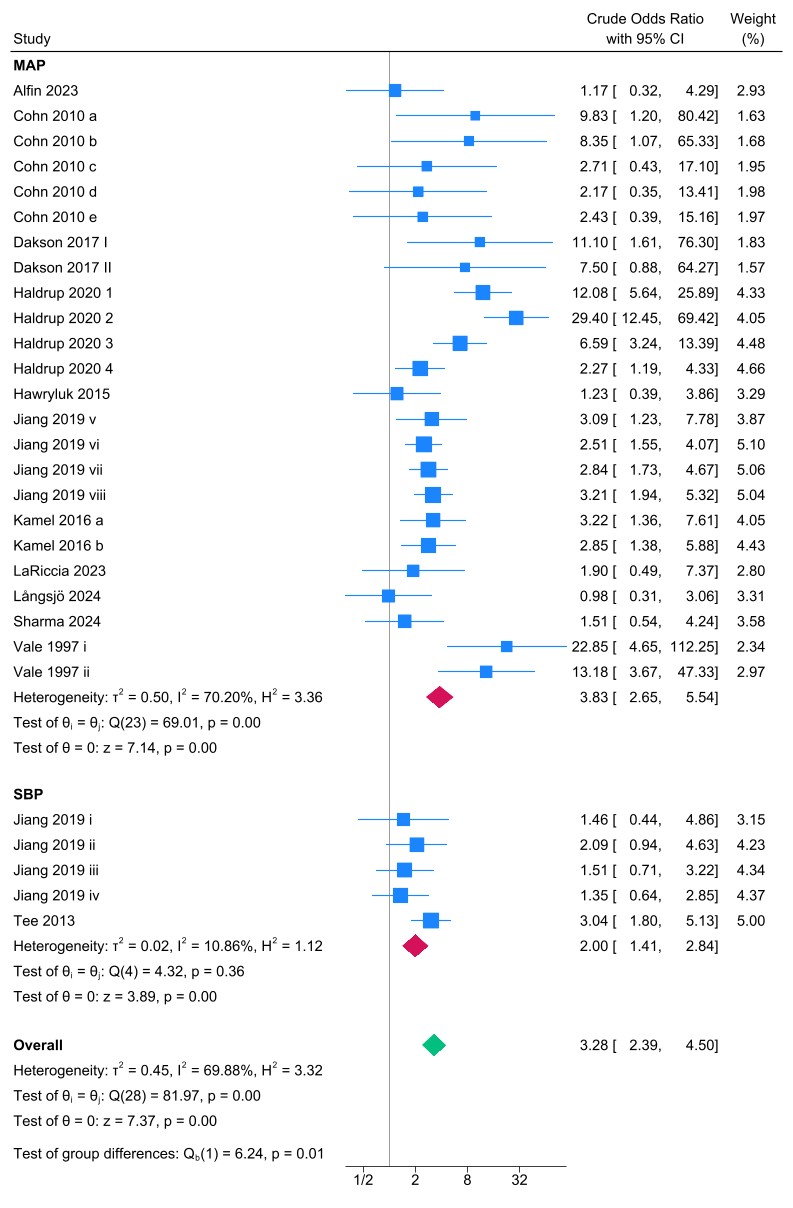


Legend: Cohn 2010 sub-studies include different MAP thresholds of a) 65mmHg, b) 70mmHg, c) 75 mmHg, d) 80mmHg, e) 85mmHg. Dakson 2017 sub-studies include different spinal levels of I) all spinal levels, II) cervical levels only. Haldrup 2020 sub-studies include different location of hypotension of 1) prehospital MAP, 2) intraoperative MAP, 3) NICU day 1-2 MAP, 4) NICU day 3-7 MAP. Jiang 2019 sub-studies include different outcomes and blood pressure type of i) association of SBP and resp function, ii) association of SBP and ambulation, iii) association of SBP and bladder function, iv) SBP and bowel function, v) association of MAP and resp function, vi) association of MAP and ambulation, vii) association of MAP and bladder function, viii) association of MAP and bowel function. Kamel 2016 sub-studies include different MAP thresholds of a) 55mmHg, b) 80mmHg. Vale 1997 sub-studies include different outcomes of a) association of MAP and no ambulatory capacity, b) association of MAP and bladder/bowel function.

### **eFigure 3:** Forest plot showing subgroup analysis by mean arterial pressure threshold (Effect estimate = unadjusted odds ratio) (29 studies)

Legend: Cohn 2010 sub-studies include different MAP thresholds of a) 65mmHg, b) 70mmHg, c) 75 mmHg, d) 80mmHg, e) 85mmHg. Dakson 2017 sub-studies include different spinal levels of I) all spinal levels, II) cervical levels only. Haldrup 2020 sub-studies include different location of hypotension of 1) prehospital MAP, 2) intraoperative MAP, 3) NICU day 1-2 MAP, 4) NICU day 3-7 MAP. Jiang 2019 sub-studies include different outcomes and blood pressure type of i) association of SBP and resp function, ii) association of SBP and ambulation, iii) association of SBP and bladder function, iv) SBP and bowel function, v) association of MAP and resp function, vi) association of MAP and ambulation, vii) association of MAP and bladder function, viii) association of MAP and bowel function. Kamel 2016 sub-studies include different MAP thresholds of a) 55mmHg, b) 80mmHg. Vale 1997 sub-studies include different outcomes of a) association of MAP and no ambulatory capacity, b) association of MAP and bladder/bowel function.

### **eFigure 4:** Forest plot showing subgroup analysis by ASIA grade injury (Effect estimate = unadjusted odds ratio) (9 studies)

### **eFigure 5:** Forest plot showing subgroup analysis by spinal level (Effect estimate = unadjusted odds ratio) (29 studies)

Legend: Cohn 2010 sub-studies include different MAP thresholds of a) 65mmHg, b) 70mmHg, c) 75 mmHg, d) 80mmHg, e) 85mmHg. Dakson 2017 sub-studies include different spinal levels of I) all spinal levels, II) cervical levels only. Haldrup 2020 sub-studies include different location of hypotension of 1) prehospital MAP, 2) intraoperative MAP, 3) NICU day 1-2 MAP, 4) NICU day 3-7 MAP. Jiang 2019 sub-studies include different outcomes and blood pressure type of i) association of SBP and resp function, ii) association of SBP and ambulation, iii) association of SBP and bladder function, iv) SBP and bowel function, v) association of MAP and resp function, vi) association of MAP and ambulation, vii) association of MAP and bladder function, viii) association of MAP and bowel function. Kamel 2016 sub-studies include different MAP thresholds of a) 55mmHg, b) 80mmHg. Vale 1997 sub-studies include different outcomes of a) association of MAP and no ambulatory capacity, b) association of MAP and bladder/bowel function.

### **eFigure 6:** Forest plot showing subgroup analysis by trauma type (isolated SCI only vs isolated and polytrauma) (Effect estimate = unadjusted odds ratio) (29 studies)

Legend: Cohn 2010 sub-studies include different MAP thresholds of a) 65mmHg, b) 70mmHg, c) 75 mmHg, d) 80mmHg, e) 85mmHg. Dakson 2017 sub-studies include different spinal levels of I) all spinal levels, II) cervical levels only. Haldrup 2020 sub-studies include different location of hypotension of 1) prehospital MAP, 2) intraoperative MAP, 3) NICU day 1-2 MAP, 4) NICU day 3-7 MAP. Jiang 2019 sub-studies include different outcomes and blood pressure type of i) association of SBP and resp function, ii) association of SBP and ambulation, iii) association of SBP and bladder function, iv) SBP and bowel function, v) association of MAP and resp function, vi) association of MAP and ambulation, vii) association of MAP and bladder function, viii) association of MAP and bowel function. Kamel 2016 sub-studies include different MAP thresholds of a) 55mmHg, b) 80mmHg. Vale 1997 sub-studies include different outcomes of a) association of MAP and no ambulatory capacity, b) association of MAP and bladder/bowel function.

### **eFigure 7:** Forest plot showing subgroup analysis by spinal cord etiology (traumatic vs non-traumatic) (Effect estimate = unadjusted odds ratio) (29 studies)

Legend: Cohn 2010 sub-studies include different MAP thresholds of a) 65mmHg, b) 70mmHg, c) 75 mmHg, d) 80mmHg, e) 85mmHg. Dakson 2017 sub-studies include different spinal levels of I) all spinal levels, II) cervical levels only. Haldrup 2020 sub-studies include different location of hypotension of 1) prehospital MAP, 2) intraoperative MAP, 3) NICU day 1-2 MAP, 4) NICU day 3-7 MAP. Jiang 2019 sub-studies include different outcomes and blood pressure type of i) association of SBP and resp function, ii) association of SBP and ambulation, iii) association of SBP and bladder function, iv) SBP and bowel function, v) association of MAP and resp function, vi) association of MAP and ambulation, vii) association of MAP and bladder function, viii) association of MAP and bowel function. Kamel 2016 sub-studies include different MAP thresholds of a) 55mmHg, b) 80mmHg. Vale 1997 sub-studies include different outcomes of a) association of MAP and no ambulatory capacity, b) association of MAP and bladder/bowel function.

### **eFigure 8:** Forest plot showing subgroup analysis by functional outcome type (Effect estimate = unadjusted odds ratio) (29 studies)

Legend: Cohn 2010 sub-studies include different MAP thresholds of a) 65mmHg, b) 70mmHg, c) 75 mmHg, d) 80mmHg, e) 85mmHg. Dakson 2017 sub-studies include different spinal levels of I) all spinal levels, II) cervical levels only. Haldrup 2020 sub-studies include different location of hypotension of 1) prehospital MAP, 2) intraoperative MAP, 3) NICU day 1-2 MAP, 4) NICU day 3-7 MAP. Jiang 2019 sub-studies include different outcomes and blood pressure type of i) association of SBP and resp function, ii) association of SBP and ambulation, iii) association of SBP and bladder function, iv) SBP and bowel function, v) association of MAP and resp function, vi) association of MAP and ambulation, vii) association of MAP and bladder function, viii) association of MAP and bowel function. Kamel 2016 sub-studies include different MAP thresholds of a) 55mmHg, b) 80mmHg. Vale 1997 sub-studies include different outcomes of a) association of MAP and no ambulatory capacity, b) association of MAP and bladder/bowel function.

### **eFigure 9:** Funnel plot (contour-enhanced) for publication bias.

Legend: Blue dots represent individual studies. Shades of grey represent different p values. Red line indicates estimated theta random effects model.

### **eFigure 10:** Galbraith Plot for exhibiting outliers.

Legend: Blue dots represent individual studies. Grey indicates 95% CI. Red line indicates regression line.
